# Experiences of adolescent girls and young women of oral PrEP uptake in rural KwaZulu-Natal

**DOI:** 10.4102/phcfm.v17i1.4952

**Published:** 2025-09-12

**Authors:** Sizwe J. Ndlovu, Siyabonga B. Dlamini, Gugulethu E. Shezi

**Affiliations:** 1 Department of Public Health Medicine, School of Nursing and Public Health, College of Health Sciences, University of KwaZulu-Natal, Durban, South Africa; 2 Cancer and Infectious Diseases Epidemiology Research Unit, College of Health Sciences, University of KwaZulu-Natal, Durban, South Africa; 3 Health Research and Knowledge Management Unit, KwaZulu-Natal Department of Health, Pietermaritzburg, South Africa

**Keywords:** PrEP experiences, adolescent health, HIV risk reduction, Richmond Municipality, pre-exposure prophylaxis, adolescent girls, young women

## Abstract

**Background:**

The human immunodeficiency virus remains a global public health concern mainly affecting adolescent girls and women. Pre-exposure prophylaxis (PrEP) uptake among this group remains low in the Richmond rural community despite known benefits.

**Aim:**

This article explores the experiences of adolescent girls and young women aged 18–24 of oral PrEP uptake in Richmond Local Municipality, KwaZulu-Natal, South Africa.

**Setting:**

The study was carried out in the peri-urban area with two traditional councils located in the uMgungundlovu district of KwaZulu-Natal, South Africa.

**Methods:**

Using qualitative methodology, 12 in-depth interviews were conducted among participants who had used PrEP between 1 month and 12 months. These interviews were thematically analysed using Colaizzi’s method.

**Results:**

Thematic analysis identified four themes capturing the experiences of adolescent girls and young women regarding PrEP uptake: (1) perceived factors influencing usage decisions, (2) barriers to uptake, (3) facilitators of continued use, (4) community education and awareness about PrEP. Barriers like stigma, healthcare access challenges and fear of side effects further hindered initiation, adherence and retention. Facilitators for continuation included family, community support and convenient access to re-supply. The study highlights the importance of school-based parental meetings and discussions to normalise PrEP use among participants in the peri-urban area.

**Conclusion:**

The identified practical approaches enable convenient resupply and could increase the uptake. Peer support is critical in improving side effect management.

**Contribution:**

The study highlights the need to improve social support by using the school’s parental meetings to educate the community about the benefits of pre-exposure prophylaxis to improve adherence and retention.

## Background

Globally, progress has been made in curbing the spread of human immunodeficiency virus (HIV) across various age groups and genders.^1^ Gender inequalities, combined with social and cultural factors, increase the vulnerability of adolescent girls and young women (AGYW) to contracting HIV.^2,3^ Young women in Eastern and Southern Africa continue to have a consistently higher prevalence of HIV compared to their counterparts, young men. In South Africa (SA), despite these gains, young women in this age group accounted for 37% of all new HIV infections in 2019.^2^ The incidence of new infections among adults has decreased from 14% to 12.7%; however, adolescents remain disproportionately affected.^4^ Human Science Research Council survey (2022) found that HIV prevalence in KwaZulu-Natal (KZN) is the second highest in SA, with an estimated 16% of the population in the province living with HIV, and AGYW contributed 9.3% to the prevalence.^5^

In KZN, uMgungundlovu District’s HIV prevalence among people aged 15–49 years stands at 24% for males and 37% for females.^6^ Multiple risk factors contribute to the high HIV incidence in AGYW, including gender-based violence (GBV), intergenerational sexual relationships, limited access to economic opportunities, and inadequate functional adolescent-friendly sexual and reproductive health services.^7,8^ Despite various HIV prevention efforts, such as pre-exposure prophylaxis (PrEP), AGYW remain highly vulnerable.

The awareness level and PrEP use among AGYW in rural KZN are significantly lower than in urban areas.^9,10^ Studies show that less than 30% of AGYW in rural settings are aware of PrEP, and even fewer understand its role in HIV prevention.^9,11,12^ The contributing factors include limited access, a lack of education and misconceptions about PrEP. In urban areas, AGYW benefit from frequent HIV prevention campaigns, increasing awareness and usage.^13^ In rural areas, barriers such as stigma, confidentiality concerns and transportation issues make accessing PrEP challenging.^14^ Cultural norms, gender dynamics and fear of judgement further discourage use.^10,15^ Thus, PrEP is often associated with promiscuity.^12,16^ The inability to consistently take PrEP pills daily because of a lack of family support exacerbates the problem of adherence and retention.^17^ Additionally, the lack of support from gatekeepers like traditional leaders and the broader community undermines PrEP acceptance.^10^ Without their endorsement, stigma and misinformation persist, and AGYW face community pressure and mistrust in healthcare services. Various studies have been conducted in South African urban and rural settings to examine adolescents’ experiences with PrEP uptake. However, there is limited knowledge of AGYW PrEP uptake experiences, adherence and continuation in farming communities, particularly in Richmond Local Municipality, KwaZulu-Natal, South Africa.

To gain a comprehensive understanding of this phenomenon, the study was based on an interpretivist paradigm and utilised the Health Belief Model (HBM) as its conceptual framework. This research explored the experiences of AGYW regarding PrEP uptake in the Richmond Local Municipality, KZN. The objectives of the study were to describe the experiences of AGYW with oral PrEP uptake in Richmond Local Municipality, South Africa; to explore the factors influencing their uptake of oral PrEP; and to examine the conditions affecting adherence and continuous use of oral PrEP among AGYW in Richmond Local Municipality, South Africa.

The identified barriers, stigma, misinformation and healthcare access challenges highlight the importance of targeted education, community awareness and family support-tailored interventions to improve adherence in similar settings where HIV prevention efforts face unique structural and social barriers.

## Research methods and design

### Study design

This qualitative study used an interpretivist paradigm to understand participants’ experiences within their context. The interpretivist perspective involves subjective epistemology and aims to comprehensively understand the phenomenon.^18,19^ An exploratory interpretive qualitative research design was employed to explore the lived experiences of adolescent girls in Richmond, focusing on enhancing PrEP uptake. We explored and understood participants’ experiences by examining how they construct identities, interpret events and convey meaning through the stories they tell. This approach allowed researchers to gain insights into AGYW’s perceptions of HIV prevalence and prevention strategies through their descriptions of these experiences.

### Setting

The study was conducted in the Richmond Local Municipality, a sub-district of the uMgungundlovu District in KZN. This municipality is predominantly a rural area located approximately 38.9 km from Pietermaritzburg, the capital city of KZN. The uMgungundlovu District had a relatively high HIV prevalence in the province, with 24% and 37% of the population aged 14–49 years affected being men and women, respectively.^6,20^ The sub-district is still recovering from the political conflict of the 1990s and has a high rate of orphans, vulnerable children and youth.^21^ Most adolescent girls complete high school, but fewer than 20% pursue higher education because of poverty, teenage pregnancy and gender norms restricting their prospects.^21^ The area is characterised by high unemployment and poverty rates, and the community has limited economic activities and poor infrastructure.^21^ Most economic activities in the region are derived from agriculture.

### Population and sampling

The study employed a non-probability, purposive sampling technique to select AGYW aged 18–24 years. The World Health Organization (WHO) defines AGYW as females aged 10–24, encompassing critical development and health vulnerability stages.^22^ The participants had used or were using PrEP for HIV prevention for at least a minimum period of 1–12 months and were residing within the community of Richmond Local Municipality. All those who are classified as males in terms of human biology were excluded from this study. A minimum of eight participants were recruited from the youth zone, local Shisa Nyama and Municipality recreational facility where youth meet to play sports and were enrolled for this study; however, data saturation determined the maximum sample size of 12.

### Data collection

The researchers conducted in-depth, face-to-face interviews in isiZulu at a community youth centre between 09 March 2024 and 12 August 2024. Given the study’s focus on discussing sexuality with young women, the inclusion of a female colleague to assist with data collection was considered appropriate and essential because the principal investigator was a male. To build rapport with possible participants, the researcher visited leisure centres and youth zones with a female colleague trained in qualitative data collection and holding a Master of Social Science degree. Following the development of rapport, study participants were sought out and enrolled. The female data collector was trained by observing the researcher conducting the in-depth interview before collecting data independently. Later, the interviews were conducted at the community youth centre. The sessions lasted an average of 17 min, with informed consent obtained from all participants, and interviews were digitally recorded. These interviews were then transcribed and translated into English. An interview guide with open-ended questions and prompts was used to explore participants’ experiences with PrEP uptake.

### Data analysis

Data collection and analysis were performed through an iterative process. The researcher transcribed the audio recordings that were in isiZulu language verbatim. After that, the transcripts were translated into English. Data analysis was guided by Colaizzi’s method of thematic analysis.^23^ This method involved repeatedly reviewing the transcripts to grasp the content (data immersion) fully, identifying key statements (coding), interpreting their meanings, and categorising the data into themes and sub-themes.^23^ Analysis was conducted with the aid of NVivo 20 software.^24^

The analysis followed an inductive approach, allowing themes to emerge naturally from the data. The researcher used bracketing through journaling to minimise bias.^25^ Recognising the potential influence of personal beliefs and biases, particularly because of the researcher’s involvement in adolescent HIV prevention initiatives, it was essential to employ strategies for mitigating these effects. The researcher utilised bracketing, a technique involving deliberately setting aside cultural background and personal experiences that could impact data interpretation. The process was supported by maintaining a reflexive journal throughout the research, complemented by regular debriefing sessions with co-authors to ensure objectivity.

### Trustworthiness

To ensure the trustworthiness of this study, the researcher employed several strategies to enhance credibility, dependability, transferability and confirmability. A trusting relationship was attempted through consistent visits to recreational facilities and youth zones, where rapport was built with potential participants to encourage true reflections on their views on the subject. The researcher was accompanied by one female colleague with a master’s degree in Social Sciences. She has sound experience in qualitative research. She conducted interviews at a familiar local youth centre, creating a safe, comfortable environment for participants to discuss their experiences with PrEP openly. The female colleague then continued with the interviews, and the researcher played the role of an observer, recording nonverbal cues displayed by the participants during the interviews.

Reflexivity and bracketing were key in maintaining objectivity, as the researcher worked in the adolescent HIV prevention sector. Bracketing minimised the influence of personal biases, ensuring that data interpretation was based on participants’ narratives. This enhanced both the objectivity and validity of the study.

Dependability was strengthened by providing a detailed account of the research process, from design to results interpretation. A codebook was developed to guide consistent data analysis, increasing the reliability of the findings. Systematic reporting of all procedures further bolstered the study’s dependability. In addition, the co-authors have extensive experience in qualitative research methods and regularly supervise qualitative research projects at both the master’s and PhD levels. Co-authors had access to the scripts for reading and data interpretation, and this facilitated triangulation. They reviewed transcripts, cross-checked data interpretations, and engaged in debriefing sessions to ensure consistency and accuracy in the emerging themes. Member checking enabled participants to confirm initial findings, ensuring their experiences were accurately represented.

Confirmability was addressed by making the transcripts available for future audits and through ongoing documentation in a research journal. This journal recorded reflections, decisions and adjustments made during the study, offering a transparent audit trail. These measures collectively enhanced the study’s trustworthiness.

### Ethical considerations

Ethical clearance to conduct this study was obtained from the University of KwaZulu-Natal Biomedical Research Ethics Committee (Reference No. BREC/00005454/2023). Permission to conduct the study in the Richmond Municipality community was also obtained from the gatekeeper and the ward councillor. Participation was voluntary, and all participants provided written informed consent before the interviews, agreeing to both the interview and the audio recording.

## Results

The study included 12 AGYW aged 18–24 years who had initiated PrEP use, as depicted in Table 1. Data saturation was reached with this sample size. Of these, four (33%) were still on PrEP, while eight (67%) had discontinued. Participants were recruited from the Richmond community, with three in high school, two employed and seven out of school. Three had children, and one disclosed living with HIV. She linked her HIV status to inconsistent use of PrEP while she was still negative, as it is a prerequisite prior to initiation. However, she discontinued use after testing positive. Four were in non-sexual relationships despite being sexually active in the past, while eight were sexually active. Pre-exposure prophylaxis use duration ranged from 1 month to 12 months.

**TABLE 1:** The profile of participants.

Participant	Age (years)	Educational level	In a relationship?	Sexually active?
P1	24	Post matric	Yes, long term	Yes
P2	18	Grade 10 high school dropout	Yes, long term	Yes
P3	21	Matriculated	No	No
P4	19	Matriculant	No	No
P5	18	Matriculant	Yes, long term	Yes
P6	22	Grade 11, high school dropout	Yes	Yes
P7	20	Matriculated	Yes, long term	Yes
P8	22	Degree	Yes, long term	Yes
P9	23	Post matric	Yes, long term	Yes
P10	19	Matriculated	No	No
P11	18	Grade 11	No	No
P12	20	Post matric	Yes	Yes

Table 2 presents the four main themes and related sub-themes emerging from the data.

**TABLE 2:** Themes and sub-themes emerged from data analysis.

Perceived factors influencing PrEP usage decisions	Barriers to PrEP uptake	Facilitators of continued PrEP use	Community education and awareness about PrEP
Knowledge of PrEP protection benefits	Challenges about access to PrEP	Family support as an enabler of PrEP use	Targeted education campaigns
Preference for condom use over PrEP	Lack of family support	Convenient re-supply options	Peer support networks
Perceptions/misconceptions	Fear of side effects	Integrate PrEP promotion into health services	Promote PrEP use for informed decision
Perceptions of a partner’s HIV status	Social stigma	-	-

PrEP, pre-exposure prophylaxis.

### Perceived factors influencing pre-exposure prophylaxis usage decisions

#### Knowledge of pre-exposure prophylaxis protection benefits

The knowledge of PrEP protection benefits seemed to affect the decision of PrEP uptake as an HIV prevention method used by AGYW. The young women were more knowledgeable about PrEP compared to adolescents. Participants pointed out that some individuals are aware of the effectiveness of PrEP:

‘… [*A*]s I have said, PrEP is the only way. I will not count condoms. In my neighbourhood, a person meets another person to have sex with on the spot, and you find they do not have protection. Like, they do not have condoms, but it’s better if the person is on PrEP.’ (P1, 24 years, post matric)

Knowledge inspired confidence to use PrEP despite not knowing the mechanics. Young women had more confidence than adolescents:

‘PrEP is a pill that protects you, even if you have sex with someone with HIV. You won’t get infected. If you take PrEP, it is not easy to get infected. I am not sure how it works, but I was told that it prevents HIV infection when they gave me at Illovu.’ (P7, 20 years, matriculated)

#### Preference for condom use over pre-exposure prophylaxis

Five out of 12 participants prefer condoms over PrEP for several reasons. Participants reported challenges with adhering to PrEP, including forgetting to take the medication and engaging in unprotected sexual activity, which heightened their risk of HIV infection. Condoms remained the popular preventative method, widely used before PrEP’s introduction. Four young women and one adolescent who identified as sexually active expressed a preference for condoms over PrEP. They disliked taking pills when not ill and harboured concerns that PrEP might harm their immune system. Condoms were perceived as more convenient and easily accessible, as they did not require frequent clinic visits. Participants who received PrEP through home or community delivery adhered to the regimen; however, when delivery ceased, their use of PrEP also declined:

‘I think it is because using condoms is easy and convenient; when you take PrEP, you have to go to certain places like the clinic to go and fetch it, you have to drink it at certain times, and there are things you are not allowed to do when you are on PrEP such as drinking alcohol, but with condoms, you can buy them or take them from the clinic, and no one will ask you anything about it.’ (P8, 22 years, degree)

‘They don’t usually use PrEP. Most prefer condoms. Others don’t like condoms, but the most popular method is condoms. As I have mentioned, with PrEP, they say it has side effects. So they are reluctant to use it because of that. Or there might be other reasons that I don’t know, but most use condoms.’ (P7, 20 years, Matriculated)

#### Perceptions and/or misconceptions

Perceptions/misconceptions about PrEP affect the decisions of participants on uptake. Young women participants had a strong sense of belief that PrEP affect their immune system, among adolescents, only one participant was uncertain about its immunological effects:

‘Most of them prefer condom use because they say that with PrEP, they do not want to take pills while they are not sick. How can you take pills to prevent getting sick? I mean, that is what they do not like: taking pills even if they are not ill. That’s what they say: do not take pills because you are not sick yet. These pills will affect the immune system because you are not sick yet.’ (P6, 22 years, high school dropout)

#### Perceptions about a partner’s HIV status

Awareness of a partner’s HIV status influenced decisions regarding PrEP use among adolescents and young women. Many participants reported discontinuing PrEP upon confirming that they and their partners were HIV-negative, perceiving it as unnecessary in such circumstances. Conversely, a lack of disclosure or uncertainty about a partner’s HIV status encouraged a more cautious approach, with some participants continuing to use PrEP until their partner’s status was confirmed:

‘… [*I*]t was like we were not trusting each other; to know his status led to me stop using PrEP. Yeah, because it prevents HIV only, and there is no need for it anymore.’ (P2, 18 years, grade 10, high school dropout)

‘First, I have to know my partner’s HIV status if he does not disclose it. I have no choice. I will be forced to check my status first; if I’m negative, I will take PrEP until I know his HIV status.’ (P7, 20 years, matriculated)

### Barriers to pre-exposure prophylaxis uptake

#### Challenges about access to pre-exposure prophylaxis

Accessibility of PrEP varied among the participants, with some finding it easy to obtain it through clinics, schools and health non-government organisations (NGOs), including home delivery services. Poor clinic treatment, including rude and judgemental behaviour from nurses, further hindered access.

Out of 12 participants, eight (adolescents and young women) said they had had negative experiences at the health facility:

‘Challenges? Yes, it is poor treatment from the clinics, and the person ends up being too lazy to collect PrEP because of lousy treatment from the clinic. They get judged when they go collect PrEP from the clinic and fear being judged that, at a young age, you are having sex.’ (P2, 18 years old, grade 10, high school dropout)

‘The thought of going to the clinic is unpleasant. Remember, it was given to us in the community. But I had to go to the clinic for re-supply and would not go to collect. Sometimes, a month would go by without the pills. I only got the month’s supply when the health NGO returned to the community.’ (P6, 22 years, grade 11, high school dropout)

#### Lack of family support

During data collection, family support was also a recurring theme. The participants mentioned that many parents lacked PrEP knowledge and did not support young people using it to protect themselves from HIV. Participants reported facing discouragement at home, with parents often questioning the necessity and effectiveness of PrEP. Families lacked information on PrEP and thought people who were on PrEP had multiple sexual partners:

‘… [*T*]he problem can be at home because it is not easy to eat it well. Parents shout at us and say that at a young age, you are having sex. You explain to them that they should get people from PrEP (Health NGO) to clarify that if you eat PrEP, it’s not that you are having sex.’ (P3, 21 years, matriculated)

A participant added:

‘I would like my family to encourage me to continue taking PrEP instead of discouraging me. They say things like, ‘Are you even sure that the pills are working or not? All you do is just taking this pill every day at 7 pm. Well, maybe you already have HIV.’ (P5, 18 years, matriculated)

Other participants were discouraged by the family members, and they ended up stopping the use of PrEP:

‘Also, my aunt told me to stop taking these pills because I was not sick. She asked why I was taking pills because I was not sick. You only take pills if you are sick, and back then, I was negative. ‘It’s better to take the pills when you are sick already’, said my aunt.’ (P6, 22 years, grade 11, high school dropout)

#### Fear of side effects

Participants had varied experiences with PrEP, with many reporting common side effects like nausea, vomiting, dizziness and headaches, while others experienced no side effects at all. The fear of potential severe side effects was common, even though some reported no side effects:

‘I experience a lot of side effects. I always feel sleepy after taking PrEP, and I experience a stomach ache. I feel dizzy, and sometimes I feel like vomiting. However, I will not stop taking PrEP because it protects me. Instead, I just take the pills when I am about to sleep.’ (P5, 18 years, matriculated)

‘What I was scared of, was the things I heard about it. People say it has terrible side effects. When I get sick, I get fragile, so I am scared of vomiting, nausea and headache. I cannot stand them. Others say you get all these side effects at the same time. So I asked myself, why should I put myself through all this? Basically, I am not sick, but I will make myself sick.’ (P7, 20 years, matriculated)

#### Social stigma

Participants highlighted the frustrating gossip surrounding PrEP use, expressing concerns about being subjected to judgement because of misinformation about its use. Young people using PrEP are often gossiped about within families and communities and incorrectly assumed to be on HIV medication or judged for being sexually active:

‘Others do not always like to take pills; they are scared people will gossip about them. It is not good to judge a person, not to make jokes about her because she is on PrEP, not to gossip about her or she becomes a topic to them.’ (P4, 19 years, matriculant)

‘But the NGO didn’t give us more than one month’s supply. When you go to the clinic, they say, ‘This one sleeps around’; hence, you are on PrEP. I remember I was asked what is problematic with abstinence. People think you are promiscuous if you are on PrEP.’ (P6, 22 years, grade 11, high school dropout)

‘Yoo, they must stop gossiping about us as people who take PrEP. They do not know about PrEP, so they think we are taking HIV medication.’ (P5, 18 years, matriculant)

### Facilitators of continued pre-exposure prophylaxis use

#### Family support as an enabler of pre-exposure prophylaxis use

Family support emerged as a critical factor influencing PrEP adherence among participants. Those who maintained PrEP usage highlighted the encouragement and assistance they received from their families. In contrast, participants who discontinued PrEP after a brief period cited a lack of family support, including active discouragement from family members, as a key reason for their non-adherence:

‘At home, they saw these pills and asked me what they were for. I told them they were for preventing HIV infection; that’s all. I used to take them every time, and they never said anything. My family was very supportive. Sometimes my mother used to remind me to take the pills.’ (P10, 19 years, matriculated)

### Community education and awareness about pre-exposure prophylaxis

#### Targeted education campaigns

The AGYW in this study were asked to share how much they knew about PrEP. It emerged that there was a sense of low levels of knowledge about PrEP and, generally, widespread misconceptions about it. Participants lacked accurate information about PrEP, and this knowledge gap extended to the broader community. This group was often exposed to misinformation that framed PrEP use negatively and, therefore, highlighted the need for education in the community:

‘If families have enough information, they can pass it on to the young women and, more importantly, encourage them to take PrEP without judging.’ (P6, 22 years, grade 11, high school dropout)

One participant further expressed the need to educate families about PrEP:

‘As I mentioned earlier, my family played a massive role in my decision about PrEP. They must be educated about it to best support their girls. If families have enough information, they can pass it on to the young women and, more importantly, encourage them to take PrEP without judging or influencing them based on inadequate information.’ (P6, 22 years, grade 11, high school dropout)

#### Peer support networks

The participants who attended the workshops on sexual reproductive health sessions in the community served as a support system for one another, and participants reported that they leaned on each other for continuous support and encouragement to continue the use of PrEP. They shared their experiences and strategies for resilience to stigma and knowledge reinforcement about PrEP:

‘They used to deliver it to us. We were attending something here in the centre with other girls, and they delivered it to us.’ (P2, 18 years, grade 10, high school dropout)

A participant raised the issue of having support from the peers:

‘They can link up with the people from PrEP to always bring it at school and in the community to link up with those who use it and have a group to support each other.’ (P3, 21 years, matriculated)

## Discussion

This study aimed to explore the experiences of AGYW aged 18–24 years in Richmond Local Municipality, South Africa, regarding the uptake of oral PrEP as a preventive measure against HIV. Consistent with study objectives, our findings offer critical insights into the multifaceted factors influencing PrEP use among this population. The results provide a nuanced understanding of how AGYW perceive, access and sustain PrEP usage, highlighting significant implications for enhancing HIV prevention strategies. This study indicates that a combination of individual knowledge, risk perception and social dynamics played a role in the PrEP initiation decision among participants. One of the key findings was that many family and community members lacked sufficient knowledge about PrEP, with some viewing it as a treatment for HIV rather than a preventive measure. This knowledge gap is consistent with findings from studies conducted in Kenya and South Africa, where a lack of awareness and misconceptions about PrEP contributed to low uptake among AGYW.^12^

Insufficient information compromised the participants’ ability to make informed decisions on HIV prevention methods, thus exacerbating their vulnerability. The results suggest that the participants opted for condom usage because of negative experiences associated with collecting re-supply from the local clinics; often, they are labelled as promiscuous because they are using PrEP. Contrary to the challenges related to accessing PrEP, condoms were easily available and did not require frequent clinic visits. Our results are different from those of Malawi, where the AGYW chose PrEP as a method of choice instead of condom use.^11^ The participants viewed the community as lacking education and awareness on PrEP and, therefore, raised the need to educate the community about PrEP so that they can receive the necessary support. These findings align with the results of a study conducted in Kenya and Uganda on how community education on PrEP increases its uptake among the general population, including AGYW.^26^ Therefore, tailored health education programmes that specifically address these gaps are crucial to empower and provide support to AGYW in the community. Such programmes should correct misconceptions and provide accurate, relatable information that empowers young women to make informed choices about HIV prevention.

The AGYW linked the decision to use PrEP to the individual’s perception of their risk of contracting HIV. The results of this study indicated that some participants stopped using PrEP when they did not perceive themselves to be at risk for HIV, particularly those in relationships with partners they trusted or who had reasons to believe their partners were HIV-negative. Participants who discontinued the use of both PrEP and condoms were at increased risk of contracting HIV and other sexually transmitted infections (STIs) and experiencing unplanned pregnancies. These findings underscore critical gaps in understanding susceptibility and vulnerability factors that contribute to the spread of HIV among adolescents and young women, similar to the results of a study conducted in Kenya and South Africa.^27^ Despite advancements in HIV prevention efforts, these results highlight the need for intensified, tailored interventions to address these challenges brought by a lack of holistic understanding of HIV risk.

Additionally, the study revealed that participants who were not sexually active discontinued PrEP after 1 month, citing the absence of sexual relationships as the primary reason for discontinuation. These findings align with prior research from PrEP demonstration projects conducted in Cape Town and Buffalo City, South Africa, as well as in Mwanza, Tanzania and Harare, Zimbabwe. These studies similarly highlighted that low perceived risk significantly undermines PrEP uptake among young African women, emphasising the critical role of risk perception in shaping adherence to HIV prevention strategies.^28^ Participants in long-term relationships with partners who had tested negative for HIV often developed trust for their partners and thus stopped using PrEP. These results reflect the HBM’s perceived susceptibility concept, where low awareness of vulnerability reduces motivation for preventive behaviours like PrEP adherence.^29^ Campaigns highlighting real-life risk factors and self-efficacy skills can encourage young women to reassess their risk and consider PrEP a proactive measure.

One of the objectives of this study was to explore the factors influencing the initiation of PrEP among AGYW. The findings highlighted several critical barriers, including stigma, limited access to healthcare services and concerns about potential side effects. Stigma emerged as a particularly prominent theme, with many participants expressing fears that using PrEP might result in being labelled as promiscuous, reflecting significant social and cultural challenges to PrEP uptake in this population. These results are consistent with the findings from a study evaluating community readiness of oral PrEP in rural South Africa, where AGYW were labelled as ‘prostitutes’, and community gatekeepers opposed the rollout.^9^ Stigma is not only a personal barrier but also a community-wide issue that affects the perception of PrEP, thereby limiting its uptake. Studies from Harare (Zimbabwe) and Johannesburg (South Africa) reported similar findings, where PrEP-related stigma is a significant barrier to uptake.^30^ These findings illustrate the deeply entrenched societal norms around sexual behaviour that can inhibit the use of HIV prevention methods.

A total of 90% of participants, across adolescents and young women, reported bad experiences when they visited the clinic to access PrEP. Both adolescents and young women participants cited negative attitudes from healthcare workers, which led them to be reluctant to visit health facilities. Our results are dissimilar to the findings of the study conducted in Cape Town, South Africa, regarding accessing clinics by young women and adolescents.^31^ These findings suggest a need for improved healthcare accessibility, which refers to addressing the negative attitude of healthcare workers, thus making it easy for AGYW to go to health facilities to get PrEP re-supplies, particularly in rural/farming settings. Mobile clinics and decentralised distribution points improve PrEP uptake by reducing the logistical challenges of accessing healthcare.^31^ The results from the study of community-based PrEP distribution indicated that convenient distribution points increase uptake. Similarly, this was observed in our study.

Concerns about side effects further hindered PrEP initiation. Participants expressed fears about the side effects of PrEP on their health. This barrier aligns with findings from a study conducted in Zimbabwe, Uganda and South Africa, which reported that the fear of side effects deterred AGYW from using PrEP.^32^ Addressing these concerns through educating AGYW with scientific, evidence-based campaigns on managing side effects and consistent communication from healthcare providers is essential. Incorporating a peer network support system in the form of peer education, where AGYW are currently using PrEP to share their experiences of managing side effects, could effectively address fears related to side effects. A study conducted in Johannesburg, South Africa, demonstrated that peer support group discussions, in which current users shared their experiences and coping strategies for mild side effects, effectively alleviated concerns and improved PrEP adherence.^33^

Additionally, informing users about the low likelihood of severe side effects and emphasising benefits outweighing risks thus increase uptake and adherence in youth. These discussions should be youth-led and occur in settings where youth congregate.^34^ This strategy fosters a supportive environment, enabling AGYW to gain confidence in managing potential side effects and enhancing their commitment to PrEP uptake.

The fear of PrEP side effects, exacerbated by misinformation, aligns with the perceived barriers component of the HBM, as these concerns deter adolescents from using PrEP, even when they recognise their susceptibility to HIV.^29^ While participants perceived PrEP as beneficial, perceived benefits did not outweigh real and perceived barriers, such as fear of side effects and social stigma.^29,35^ Contrary to counselling, the peer education programme is suitable for AGYW, where trained young women serve as ambassadors to share relatable experiences of mitigating PrEP side effects, which may also help improve uptake.

The study also sought to examine the conditions that facilitate adherence and continuous use of PrEP among AGYW. We found that participants with strong family support reported they were likely to continue using PrEP, indicating that supportive relationships are critical in PrEP retention. Contrary to our findings, the results of the study from uMkhanyakude, South Africa involving young women who sell sex showed that the participants continued with PrEP despite the lack of family and community support.^10^ In an efficacy study conducted in Zimbabwe and Kenya, results indicated the effect of social support as an enabler of PrEP adherence; these findings are consistent with the results of our study.^30^ Participants with easy access to PrEP refills and regular follow-up were likely to adhere to the medication regimen. This is consistent with the study conducted in the Western Cape, South Africa, highlighting convenient re-supply as a critical factor in adherence to PrEP for the AGYW.^31^ This underscores the importance of decentralising the distribution of PrEP re-supply and devising innovative ways to reach AGYW in rural areas.

Positive reinforcement from caregivers or parents also played a significant role in encouraging PrEP continuation. Study participants who received positive feedback about their commitment to HIV prevention from parents/caregivers felt more motivated to continue using PrEP. This emphasises the need for caregivers to offer ongoing encouragement and praise to adolescents for using PrEP. Strong support systems and positive reinforcement could act as cues to action, boosting adolescents’ self-efficacy, which empowers them to confidently adhere to and continue using PrEP as outlined in the HBM.^29^

The findings from this study also highlight the critical role of community education and awareness in shaping attitudes towards PrEP. In Richmond Local Municipality, the perceived low levels of community knowledge about PrEP seemed to have contributed to the stigma and misconceptions surrounding its use. Comprehensive community education campaigns that reach adolescents and the broader population are necessary to normalise PrEP use.^9^ The results align with a study conducted in Zimbabwe and Uganda, which found that increased community knowledge of PrEP led to improved uptake.^32^ These campaigns should target community leaders, for example, traditional leaders and healers to ensure widespread dissemination of accurate information in peri-urban areas. However, these findings were based on a study with a small sample size. Representative studies with larger sample sizes are recommended to measure the extent of this challenge.

School parental meetings can serve as neutral platforms for PrEP education to strengthen parental and family involvement.^36^ Incorporating PrEP into parenting discussions and broader health agendas equips families and communities with the knowledge to support adolescents in making informed HIV prevention choices.^33^ The initiative may also help normalise PrEP use by framing it as an integral part of sexual health education, reducing stigma and fostering acceptance.^37^ Finally, peer educators and community health workers are crucial in spreading accurate information about PrEP and supporting adolescents in decision-making.^31,38^ Empowering these groups through training and resources can help ensure that PrEP information reaches even the most marginalised and hard-to-reach communities in Richmond Local Municipality.

Social stigma was another significant barrier highlighted by the participants. In our study, we found that AGYW faced judgement and gossip about their sexual activity and pill usage, particularly in community and clinic settings. Participants described that social stigma and gossip within communities emerged as substantial barriers to PrEP use. Lessons from community inclusion, including community ‘leaders’ studies in Kenya, South Africa and Nigeria, indicate the need for community buy-in for the successful rollout of PrEP.^9,26,39,40^

Poor treatment at clinics, including rude and judgemental behaviour from healthcare workers, was frequently mentioned as a barrier. We found that participants felt stigmatised and judged for using PrEP, with some being labelled promiscuous or facing intrusive questions about their sexual activity, which discouraged them from continuing PrEP use. This study highlights the complex factors influencing PrEP uptake and continuation among AGYW in Richmond Local Municipality, South Africa. Addressing the barriers to PrEP initiation, such as stigma and healthcare access, while reinforcing facilitators of adherence, such as support systems and positive reinforcement, is essential for improving PrEP outcomes in this population. Functional youth-friendly healthcare services are critical components of any strategy to increase adolescent PrEP uptake and adherence. Addressing these factors holistically can significantly enhance HIV prevention efforts in Richmond Municipality and similar settings.

Qualitative studies play a vital role in complementing quantitative research by offering a deeper understanding of the challenges derived from lived experience surrounding PrEP uptake among AGYW. This study’s strength lies in its rigorous design, data collection and analysis, which provide detailed insights into AGYW’s real-life experiences using PrEP. To our knowledge, no other studies have explored PrEP uptake experiences among AGYW in the Richmond Local Municipality, KwaZulu-Natal. Unlike previous research, our study identifies specific factors that support PrEP use among AGYW in a farming community (Richmond Local Municipality).

### Study limitations

Several limitations impact the findings of the study. Because it was confined to one community, it could not be applied to other areas. Concentrating on AGYW’s perspectives and not considering views from other groups, like men or older women, makes the findings not generalisable to other regions. Although data saturation was achieved, a larger sample size could have yielded more diverse perspectives. The study did not compare views across age groups or fully analyse how the PrEP programme was implemented. Self-reported data were used exclusively, and it may be biased in the data reported by participants.

### Recommendations for future studies

Findings suggest a need for a study with a larger sample to examine the community of Richmond Local Municipality, including community gatekeepers’ understanding of PrEP, and determine whether there is a general negative attitude towards AGYW using this prevention method in the community at large that contributes to low uptake. A range of demographics, including older women and men, should be included in future studies to have a deeper knowledge of PrEP uptake in these populations. To improve generalisability, the study should also examine AGYW’s experiences in other socio-cultural and geographic contexts. Mixed methods and data triangulation can increase reliability and yield fresh insights. Studies should also assess interventions such as community-based PrEP distribution and educational initiatives to improve adherence and retention. Persistent PrEP use and evolving barriers can be examined through longitudinal research. Innovative strategies like peer support groups and digital health technologies should be developed to improve adherence and reduce stigma in settings with limited resources.

## Conclusion

This study explored the perceptions of AGYW and broadened the understanding of why the PrEP uptake is significantly low among this population in Richmond, despite the knowledge of its benefits based on their views. The study captured the voices of the AGYW’s experiences in accessing HIV prevention commodities. Furthermore, practical approaches to increasing uptake by removing barriers and improving facilitators that make PrEP more accessible were studied. The study highlighted the need for targeted education efforts to reduce stigma and raise community understanding about PrEP. By removing barriers through conventional leadership training and community-focused interventions, PrEP uptake and adherence among AGYW in rural regions can be improved.
